# Factors Affecting the Ability of the Spectral Domain Optical Coherence Tomograph to Detect Photographic Retinal Nerve Fiber Layer Defects

**DOI:** 10.1371/journal.pone.0116115

**Published:** 2014-12-23

**Authors:** Harsha L. Rao, Uday K. Addepalli, Ravi K. Yadav, Nikhil S. Choudhari, Sirisha Senthil, Chandra S. Garudadri

**Affiliations:** VST Glaucoma Center, L V Prasad Eye Institute, Banjara Hills, Hyderabad, India; Casey Eye Institute, United States of America

## Abstract

**Purpose:**

To evaluate the ability of normative database classification (color-coded maps) of spectral domain optical coherence tomograph (SDOCT) in detecting wedge shaped retinal nerve fiber layer (RNFL) defects identified on photographs and the factors affecting the ability of SDOCT in detecting these RNFL defects.

**Methods:**

In a cross-sectional study, 238 eyes (476 RNFL quadrants) of 172 normal subjects and 85 eyes (103 RNFL quadrants with wedge shaped RNFL defects) of 66 glaucoma patients underwent RNFL imaging with SDOCT. Logistic regression models were used to evaluate the factors associated with false positive and false negative RNFL classifications of the color-coded maps of SDOCT.

**Results:**

False positive classification at a p value of <5% was seen in 108 of 476 quadrants (22.8%). False negative classification at a p value of <5% was seen in 16 of 103 quadrants (15.5%). Of the 103 quadrants with RNFL defects, 64 showed a corresponding VF defect in the opposite hemisphere and 39 were preperimetric. Higher signal strength index (SSI) of the scan was less likely to have a false positive classification (odds ratio: 0.97, p = 0.01). Presence of an associated visual field defect (odds ratio: 0.17, p = 0.01) and inferior quadrant RNFL defects as compared to superior (odds ratio: 0.24, p = 0.04) were less likely to show false negative classifications.

**Conclusions:**

Scans with lower signal strengths were more likely to show false positive RNFL classifications, and preperimetric and superior quadrant RNFL defects were more likely to show false negative classifications on color-coded maps of SDOCT.

## Introduction

Glaucoma is a progressive optic neuropathy characterized by typical optic disc and retinal nerve fiber layer (RNFL) changes, with or without visual field (VF) defects. RNFL loss is a very early clinical sign of glaucoma, which is reported to be present in majority of glaucoma patients much before any detectable VF defects. [1], [2] Red free fundus photography is currently considered the gold standard for RNFL examination and loss of RNFL is seen as a wedge shaped defect in arcuate pattern, the width of which is larger than the major blood vessel, and reaching the edge of the disc margin. [3] Other techniques available for RNFL evaluation are scanning laser polarimetry and optical coherence tomography (OCT). Spectral domain OCT (SDOCT) is a recent generation of OCT technology which has shown good ability to detect RNFL defects in glaucoma. [4]–[8] However, the ability of SDOCT to detect RNFL defects is influenced by multiple factors. These factors can in general be disease-related, subject- or eye-related and test- or technology-related. Disease-related factor known to influence the ability of SDOCT to detect RNFL defects is the size of the defect. Ability of SDOCT is shown to be better in detecting wider compared to narrower RNFL defects. [4]–[8] Eye-related factors reported to influence the ability of SDOCT to detect RNFL defects are the optic disc size and axial length of the eye. [8], [9] One of the technology-related factors evaluated, but found not to influence the ability of SDOCT to detect RNFL defects is the signal strength of the scan [8].

As SDOCT is non-patented technology, various manufacturers have their own SDOCT devices. Though different SDOCT devices provide different maps for RNFL evaluation, one map common to all SDOCT devices is the color-coded map, which compares the RNFL thickness of a subject with the age and ethnicity-matched normative database within the machine. The ability of SDOCT to detect RNFL defect on color-coded map is likely to depend on the robustness of the internal normative database composition. However there are no studies evaluating the ability of SDOCT in correctly identifying clinically detectable RNFL defects in an Indian population. The aim of this study was to evaluate the ability of the internal normative database classification of SDOCT in detecting wedge shaped RNFL defects identified on photographs in an Indian population and the covariates affecting the ability of SDOCT in detecting these RNFL defects.

## Methods

This was an observational, cross-sectional study of consecutive subjects referred by general ophthalmologists to a tertiary eye care facility between September 2010 and November 2012 for a glaucoma evaluation. All subjects were of Indian origin. Written informed consent was obtained from all subjects to participate in the study and the Institutional Review Board of L V Prasad Eye Institute approved the methodology. All methods adhered to the tenets of the Declaration of Helsinki for research involving human subjects.

Inclusion criteria were age ≥18 years, best corrected visual acuity of 20/40 or better and refractive error within ±5 D sphere and ±3 D cylinder. Exclusion criteria were presence of any media opacities that prevented good quality optic disc photographs and SDOCT imaging, and any retinal (including macular) disease other than glaucoma which could confound the evaluations. All participants underwent a comprehensive ocular examination which included a detailed medical history, best corrected visual acuity measurement, slit-lamp biomicroscopy, Goldmann applanation tonometry, gonioscopy, dilated fundus examination, standard automated perimetry (SAP), digital optic disc photography and SDOCT imaging with RTVue (Optovue Inc, Fremont, CA).

SAP was performed using a Humphrey Field analyzer, model 750i (Zeiss Humphrey Systems, Dublin, CA), with the Swedish interactive threshold algorithm (SITA) standard 24-2 algorithm. Visual fields (VF) with fixation losses, false positive and false negative response rates of less than 20% were considered reliable. VFs were considered glaucomatous if the pattern standard deviation had a P value of less than 5% and the glaucoma hemifield test result was outside normal limits. [10] In cases of glaucomatous VF, the hemisphere that contained the VF defect was noted down. VF defect was defined as a cluster of 3 points with probabilities of <5% on the pattern deviation probability map in 1 hemifield, including 1 or more points with a probability of <1%.

Digital optic disc photographs were obtained by trained technicians (FF 450plus with Visupac 4.2.2, Carl Zeiss Meditec Systems GmbH, Pirmasens, Germany). Photographs consisted of a 50 degree image centered on the optic disc, a similar image centered on the macula, a 30 degree image centered on the optic disc and a 20 degree image centered on the disc. All these images also consisted of one colored and one red-free image each. Each photograph was evaluated by two of the four experts (HLR, UKA, NSC and SS) independently, who were masked to the clinical examination results of the subjects and also the results of visual field, imaging and other eye examination results. Experts graded the presence or absence of the following features on disc photographs: superior and inferior neuroretinal rim thinning, superior and inferior rim notch, superior and inferior disc hemorrhage, and superior and inferior wedge shaped RNFL defect. Any of these findings which the experts were not sure about were graded as “suspicious”. Discrepancies between the two experts were resolved by consensus. Eyes, where a consensus could not be reached, were excluded from analysis. Eyes where a feature was graded as suspicious by either of the experts were also excluded from the analysis. Experts also made an overall classification of glaucoma and non-glaucoma based on the above features. Eyes, where a classification to either glaucoma or non-glaucoma group was not possible by either or both the experts (true disc suspects), were also excluded from the analysis.

SDOCT examination was performed with the RTVue (software version 5.1.0.90). RTVue uses a scanning laser diode with a wavelength of 840±10 nm to provide images of ocular microstructures. The ONH (optic nerve head) protocol was used for RNFL imaging with RTVue in this study. This protocol has been explained earlier. [11], [12] RNFL thickness around the optic disc is divided into eight 45 degree sectors and each is compared with the age and ethnicity-matched internal normative database within the software and one of the three diagnostic categorizations (color-code) is provided. “Outside normal result” categorization (red-code) indicates that the value is lesser than the lower 99% confidence interval (CI) of the healthy, ethnicity- and age-matched population. “Borderline” result (yellow-code) indicates that the value is between the 95% and 99% CI, and a “within-normal-limits” (green-code) indicates that the value is within the 95% CI. RTVue is the only commonly used SDOCT device that has a sizeable Indian normative database within it. The internal normative database of RTVue SDOCT consists of 861 normal eyes of which 119 are of Indian origin. The diagnostic categorizations of the superotemporal and inferotemporal RNFL sectors of SDOCT were noted down. Scan quality of the SD-OCT image was based on the signal strength index (SSI). SSI is a proprietary measure of the average signal strength across the scan. The SSI can range from 0 (no signal) to 100 (very strong signal). The stronger the OCT signal, the higher the SSI. As per the manufacturers’ guidelines, all scans with a SSI score of <30 were excluded from the analysis. Eyes in which the segmentation algorithm failed were also excluded. All patients had disc photography, VF testing and SDOCT examination performed on the same day.

## Statistical Analysis

Descriptive statistics included mean and standard deviation for normally distributed variables and median and inter-quartile range (IQR) for non-normally distributed variables.

Diagnostic categorization of superotemporal and inferotemporal RNFL sectors were analyzed against the presence or absence of RNFL defects in the corresponding regions on photographs. Each RNFL quadrant (superotemporal and inferotemporal) was considered as a unit for this analysis. Logistic regression models were used to evaluate the factors associated with false positive and false negative RNFL classifications of SDOCT. As measurements from both eyes of the same subject are likely to be correlated, the standard statistical methods for parameter estimation lead to underestimation of standard errors and to confidence intervals that are too narrow. [13] Therefore, the cluster of data for the subject was considered as the unit of resampling and the standard errors were adjusted using clustered sandwich estimation method. Statistical analyses were performed using commercial software (Stata ver. 11.2; StataCorp, College Station, TX).

## Results

**Figure 1:**
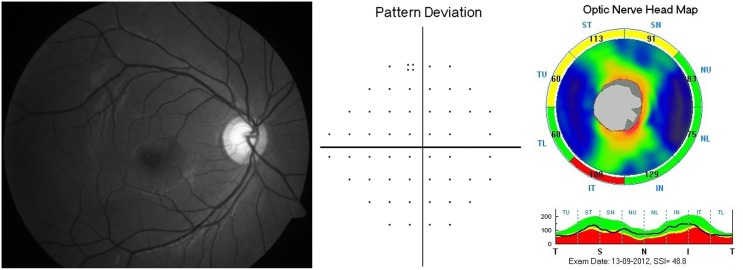
Example of a false positive classification on color-coded map of spectral domain optical coherence tomograph (SDOCT). Left panel shows the red free fundus photograph of an eye with healthy retinal nerve fiber layer (RNFL). Middle panel shows normal pattern deviation probability plot of the same eye. Right panel shows the color-coded RNFL map of SDOCT showing abnormal RNFL both in superotemporal (ST, at p<5%) and inferotemporal (IT, at p<1%) quadrants.

**Figure 2:**
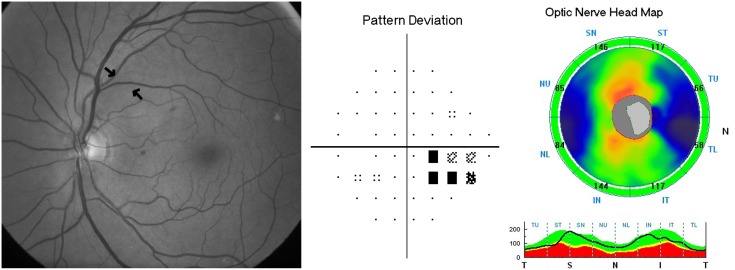
Example of a false negative classification on color-coded map of spectral domain optical coherence tomograph (SDOCT). Left panel shows the red free fundus photograph of an eye with superotemporal retinal nerve fiber layer (RNFL) defect. Middle panel shows the corresponding visual field defect in the inferior hemisphere on the pattern deviation probability plot of the same eye. Right panel shows the color-coded RNFL map of SDOCT showing normal RNFL in superotemporal (ST) quadrant.

Six hundred and seventy eight eyes of 382 consecutive subjects referred for glaucoma evaluation to our center were analyzed. Forty two eyes with unreliable VFs and 7 eyes with poor quality disc photographs were excluded. Further, 12 eyes with segmentation algorithm failure and 10 eyes with SSI<30 on ONH scans were excluded. Of the remaining eyes, 71 eyes classified as either optic disc or RNFL suspect by either or both the experts, and 51 eyes with optic disc classification as normal and VF classification as glaucoma were also excluded, leaving 485 eyes for the current analysis. Of these, 238 eyes of 172 subjects with optic disc, RNFL and VF classification as “non-glaucoma” formed the control group. Of the remaining 247 eyes with optic disc classification as glaucoma, localized RNFL defects were identified by experts in 85 eyes of 66 subjects, which formed the case group. The initial agreement between glaucoma experts for the overall optic disc classification was 92.7% (kappa = 0.63). The initial agreement between experts for RNFL defect identification was 89.2% (kappa = 0.42). Remaining RNFL defect identification was by consensus. Table 1 shows the demographic, spherical equivalent refraction, VF and SDOCT parameters of the two groups. All VF parameters were significantly different in the glaucoma compared to the control group. Glaucoma patients had significantly smaller optic discs than the control subjects. SSI values were comparable between the two groups. Two hundred and thirty eight control eyes contributed 476 RNFL quadrants (238 superior and 238 inferior) and 85 glaucoma eyes contributed 103 RNFL quadrants with defects (56 superior and 47 inferior RNFL defects) for the analysis. Table 2 shows the diagnostic categorization of the corresponding RNFL quadrants on SDOCT. False positive classification at a p value of <1% was seen in 31 of 476 quadrants (6.5%) and at a p value of <5% was seen in 108 of 476 quadrants (22.8%). False negative classification at a p value of <1% was seen in 31 of 103 quadrants (30.1%) and at a p value of <5% was seen in 16 of 103 quadrants (15.5%). Figs 1 and 2 show examples of false positive and negative RNFL defect classifications respectively on the color-coded maps of SDOCT. Table 3 shows the diagnostic categorization of the corresponding RNFL quadrants on SDOCT separately in glaucoma eyes with and without corresponding VF defects. SDOCT identified the RNFL defect better when the RNFL defect was associated with a corresponding VF defect (p = 0.004, Chi square test). Of the 103 quadrants with RNFL defects, 64 showed a corresponding VF defect in the opposite hemisphere and 39 RNFL defects were preperimetric.

**Table 1:** Age, spherical equivalent refraction, visual fields and spectral domain optical coherence tomographic characteristics of the participants.

	Control group(238 eyes of 172 subjects)	Glaucoma group(85 eyes of 66 patients)	P value
**Age (years)**	54 (45, 62)	54 (47, 61)	0.57
**Spherical equivalent (diopter)**	0 (–1, 1)	0 (–1.5, 0.75)	0.10
**Mean deviation (dB)**	–1.8 (–3.1, −0.7)	–4.7 (–6.9, −2.2)	<0.001
**Pattern standard deviation (dB)**	1.7 (1.5, 2.0)	3.4 (2.1, 8.4)	<0.001
**Visual field index (%)**	99 (98, 99)	93 (83, 97)	<0.001
**Optic disc area (mm^2^)**	2.2 (1.8, 2.6)	1.9 (1.6, 2.3)	0.001
**Signal strength index**	51 (45, 59)	52 (45, 57)	0.28

dB: decibel. All values are median and inter-quartile ranges.

**Table 2:** Diagnostic categorization of retinal nerve fiber layer (RNFL) parameter on spectral domain optical coherence tomograph (SDOCT) in eyes with and without RNFL defects on photographs.

RNFL defect on photograph	Diagnostic categorization of RNFL on SDOCT		
	WNL	Borderline	ONL
Absent	368	77	31
Present	16	15	72

WNL: within normal limits; ONL: outside normal limits.

**Table 3:** Presence of corresponding defect on visual field and the diagnostic categorization of retinal nerve fiber layer (RNFL) parameter on spectral domain optical coherence tomograph (SDOCT) in eyes with RNFL defects on photographs.

Presence of visual field defect	Diagnostic categorization of RNFL on SDOCT		
	WNL	Borderline	ONL
Yes	5	7	52
No	11	8	20

WNL: within normal limits; ONL: outside normal limits.

**Figure 3:**
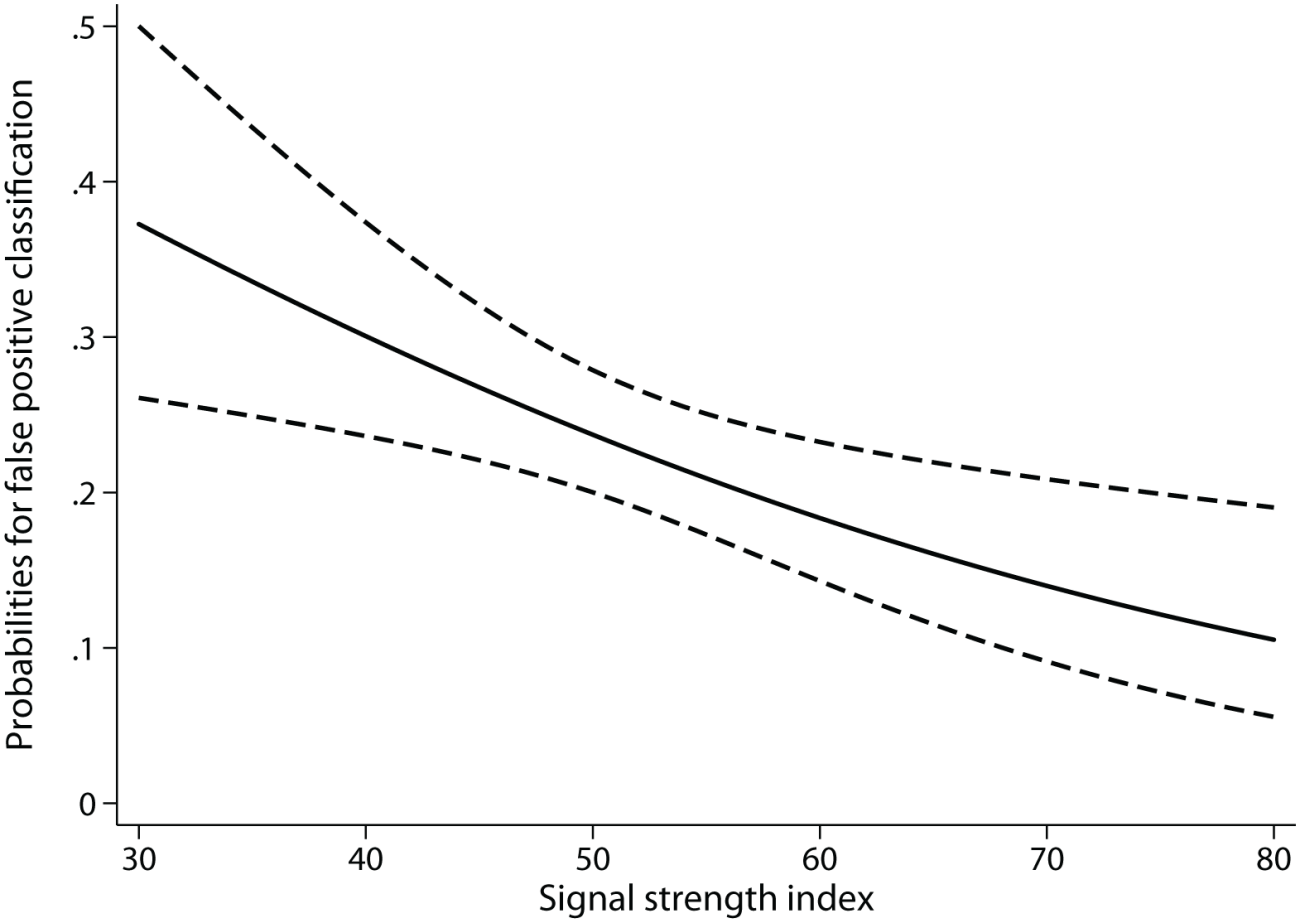
Predicted probability of false positive classification of retinal nerve fiber layer defects on spectral domain optical coherence tomograph with different signal strength index values.

Table 4 shows the results of the logistic regression models evaluating the odds ratios for the factors associated with false positive and false negative RNFL classifications on SDOCT. “Borderline” category of RNFL classification on SDOCT was clubbed with the “outside normal limits” category for this analysis. SSI was significantly associated with false positive classifications indicating that scans of higher SSI value were less likely to have false positive classification. Fig. 3 shows the predicted probability of false positive RNFL classifications at different SSI values according to the logistic regression model. Probability of false positive classifications, which was 10% (95% CI: 5–19) at a SSI value of 80 increased to 24% (20–28) at a SSI value of 50 and 37% (26–50) at a SSI value of 30. Presence of a corresponding VF defect was significantly associated with a lower probability of false negative classification. Probability of a false negative classification when a RNFL defect was associated with a corresponding VF defect was 8% (3–17), while the same when a RNFL defect was preperimetric was 31% (18–47). Table 4 also shows that superior quadrant RNFL defects were more likely to have false negative RNFL classification on SDOCT. Probability of false negative classification in cases of inferior quadrant RNFL defects was 12% when associated with a corresponding VF defect and 39% when preperimetric. The same in cases of superior quadrant RNFL defects was 36% and 64% respectively.

**Table 4:** Factors causing misclassification of retinal nerve fiber layer (RNFL) parameters on spectral domain optical coherence tomograph (SDOCT).

	False positiveclassification			False Negativeclassification		
Parameter	Odds ratio	95% CI	P value	Odds ratio	95% CI	P value
Signal strength index	0.97	0.94, 0.99	0.01	0.97	0.92, 1.02	0.23
Disc area	1.29	0.83, 2.00	0.26	0.63	0.21, 1.87	0.40
Spherical equivalent	1.00	0.86, 1.17	0.97	1.10	0.84, 1.44	0.48
RNFL quadrant(superior as reference)	1.16	0.79, 1.69	0.45	0.24	0.06, 0.92	0.04
Presence of VF defect				0.18	0.05, 0.65	0.01

CI = confidence interval; VF: visual field.

The results were similar when we clubbed the “borderline” category of RNFL classification on SDOCT with the “within normal limits” category. Scans with higher SSI values, still were less likely to have false positive classifications (odds ratio: 0.96, p = 0.06), and perimetric RNFL defects were less likely to have false negative RNFL classifications (odds ratio: 0.31, p = 0.01) on SDOCT. Inferior quadrant RNFL defects were less likely to show false negative classifications (odds ratio: 0.39, p = 0.07). We also ran separate models considering one RNFL quadrant per eye and one eye per subject for analysis; the results were essentially the same as above.

## Discussion

In this study to evaluate the misclassification rates of the internal normative database classification of RTVue in detecting photographic wedge shaped RNFL defects in an Indian population, we found that the false positive rate was 22.8% and the false negative rate was 15.5% at a p value of <5%.

Ability of the internal normative database of a test to detect disease depends on the robustness of its composition. If the normal population is well represented with respect to all its attributes, within the normative database, the ability of normative database to correctly identify normal and diseased groups is good. SDOCT is a commonly used imaging device in glaucoma currently and various manufacturers have their own commercially available SDOCT device. RTVue is one such SDOCT and is one of the very few with a sizeable ethnicity-specific normative database, consisting of 119 eyes from Indian population. Though there are no earlier studies evaluating the ability of the internal normative database of RTVue SDOCT in detecting RNFL defects in Indian population, a similar study in Japanese population has reported the false positive and negative classification rates to be 26.7% and 15.6% respectively. [7] This is very similar to the results of our study. It is important to note that the normative database of RTVue SDOCT also consists of 160 eyes of Japanese ethnicity. Similar studies in Korean population using a different SDOCT device (Cirrus HD-OCT; Carl Zeiss Meditec Inc, Dublin, CA) have reported the false positive classification rates to be between 10% and 20% and the false negative classification rates to be between 10% and 50% [4], [5], [8], [9].

Understanding the factors affecting the performance of a diagnostic test is important in order to evaluate the applicability of a test under different clinical scenarios. Evaluating the factors associated with the misclassification of RNFL parameters on SDOCT, we found that the signal strength was significantly associated with false positive classification, with scans of lower SSI more likely to show false positive classifications. We have earlier reported that the diagnostic abilities (in terms of area under the receiver operating characteristic curves, AUC) of RNFL parameters of SDOCT in glaucoma decreased with decrease in signal strength. [14] Current study shows that the decrease in AUCs of RNFL parameters with decreasing signal strength is in fact due to an increase in the false positive classification rate that is seen with lower signal strengths. The reason for false positive classifications is probably because of the underestimation of RNFL thickness with lower signal strengths, as has been reported by Wu et al using an earlier version of OCT. [15] However a similar association was not found in a previous study by Hwang et al that evaluated the association between false positive RNFL classification and signal strength. [8] This contrary result may be related to the difference in the way signal strength of the SDOCT scan was assessed in our study and in the study by Hwang et al. [8] Signal strength assessment is based on proprietary scales which may not be comparable between different SDOCT devices. For example, the SSI of RTVue, the SDOCT used in our study, ranges from 0 to 100, while that of Cirrus HD-OCT, used in the study by Hwang et al, ranges from 0 to 10. As per the manufacturer’s recommendations, we included all scans with a SSI value of over 30 and Hwang et al included all scans with signal strength of over 6.

Previous studies have reported an association of false positive classification on SDOCT with longer axial length. [8], [9] Few previous studies have also shown thinner RNFL measurements with SDOCT in myopic eyes. [16], [17] We did not find such an association between spherical equivalent refraction and false positive RNFL classification in our study. This is probably because of the narrow range of refractive errors (within ±5 diopters) and limited number of myopic eyes in our study. Studies by Kim et al and Hwang et al, unlike our study, included a wider range of myopic refractive errors; up to −8 diopters in the study by Kim et al [9] and −11 diopters in the study by Hwang et al [8] and were therefore better powered to evaluate this association.

Previous studies have also reported an association of false positive classification on SDOCT with smaller disc size. [9] We again did not find such an association. It is important to however note that the disc size measurements were performed on SDOCT in our study but was performed on time domain OCT in the study by Kim et al [9] and previous studies have shown that the disc size measurements between the two OCT technologies are not comparable [18], [19].

Evaluating the factors associated with false negative classification of RNFL defects on SDOCT, we found that preperimetric RNFL defects were associated with significantly higher false negative classification. Size of the RNFL defect has been reported to be associated with false negative classification on SDOCT in multiple previous studies. [4]–[8] This has been reported to be because of the masking of small RNFL defects while averaging over a sector or because of the defect falling between 2 adjacent sectors. We also found an association between false negative RNFL classifications on SDOCT and superior quadrant RNFL defects. The reason for this finding however is not clear. More preperimetric RNFL defects were seen in superior quadrant (23/39) compared to inferior quadrant (16/39). However, even after adjusting for the presence of corresponding visual field defect, the odds of false negative classification was significantly higher for superior quadrant RNFL defects.

A limitation of our study is the exclusion of eyes with diffuse RNFL loss from the analysis. The ability of the color-coded RNFL map of SDOCT to identify diffuse RNFL loss may be different from that of the SDOCT to detect localized wedge shaped RNFL defects. Therefore the results of our study may not be applicable to eyes with diffuse RNFL loss. An important thing to note here is that the control group in our study was selected from the group of subjects referred as glaucoma suspects based on their optic disc appearance by general ophthalmologists. Therefore, a possible limitation of our study is the inclusion of a few preperimetric glaucoma cases into the control group which might have increased the false positive rate of the RNFL classification of SDOCT. This is however less likely as two glaucoma experts independently identified the optic discs as non-glaucomatous and the RNFL to be normal. There was no ambiguity in their classification by the glaucoma experts. Therefore in true sense, optic discs included in the control group though were referred as suspects for glaucoma, were not true suspects but were large discs with large physiological cups that caused a diagnostic uncertainty among general ophthalmologists. We excluded such true suspects (optic discs which were unable to be classified into glaucoma or non-glaucoma group, by one or both of the experts and RNFL where the presence or absence of a defect was suspicious) from the analysis. Such true suspects would require a longitudinal study to look for progressive structural changes and to definitively classify them into glaucoma or non-glaucoma groups. [20] We believe that including a control group which is likely to cause some amount of diagnostic uncertainty is more meaningful and mimics the real-life clinical situation than a control group with no suspicious findings of the disease as was used in the previous studies. We have earlier used such a control group for evaluating the diagnostic ability of imaging technologies in glaucoma [12], [14], [21], [22].

In conclusion, clinicians need to be mindful of the false classification rates of photographic RNFL defects on the color-coded probability maps of SDOCT. In this study, scans with lower signal strength were associated with higher false positive classifications, and preperimetric and superior quadrant RNFL defects were associated with higher false negative classifications on color-coded maps of SDOCT. Clinicians should take these factors into account while evaluating the color-coded RNFL maps of SDOCT.
